# Red peppers with moderate and severe pungency prevent the memory deficit and hepatic insulin resistance in diabetic rats with Alzheimer’s disease

**DOI:** 10.1186/s12986-015-0005-6

**Published:** 2015-03-08

**Authors:** Hye Jeong Yang, Dae Young Kwon, Min Jung Kim, Suna Kang, Na Rang Moon, James W Daily, Sunmin Park

**Affiliations:** Division of Metabolism and Functionality Research, Korean Food Research Institutes, Sungnam, South Korea; Department of Food and Nutrition, Obesity/Diabetes Research Center, Hoseo University, 165 Sechul-Ri, BaeBang-Yup, Asan-Si, ChungNam-Do 336-795 South Korea; Daily Manufacturing Inc., Rockwell, NC USA

**Keywords:** Red pepper, Pungency, Alzheimer’s disease, Insulin resistance, Insulin secretion

## Abstract

**Background:**

Dementia induced by β-amyloid accumulation impairs peripheral glucose homeostasis, but red pepper extract improves glucose homeostasis. We therefore evaluated whether long-term oral consumption of different red pepper extracts improves cognitive dysfunction and glucose homeostasis in type 2 diabetic rats with β-amyloid-induced dementia.

**Methods:**

Male diabetic rats received hippocampal CA1 infusions of β-amyloid (25–35) (AD) or β-amyloid (35–25, non-plaque forming), at a rate of 3.6 nmol/day for 14 days (Non-AD). AD rats were divided into four dietary groups receiving either 1% lyophilized 70% ethanol extracts of either low, moderate and severe pungency red peppers (AD-LP, AD-MP, and AD-SP) or 1% dextrin (AD-CON) in Western diets (43% energy as fat).

**Results:**

The ascending order of control < LSP < MSP and SSP potentiated the phosphorylation of CREB and GSK and inhibited Tau phosphorylation in the hippocampus which in turn inhibited β-amyloid accumulation. The inhibition by MP and SP reduced the memory deficit measured by passive avoidance test and water maze test. Furthermore, the accumulation of β-amyloid induced glucose intolerance, although serum insulin levels were elevated during the late phase of oral glucose tolerance test (OGTT). All of the red pepper extracts prevented the glucose intolerance in AD rats. Consistent with OGTT results, during euglycemic hyperinulinemic clamp glucose infusion rates were lower in AD-CON than Non-AD-CON with no difference in whole body glucose uptake. Hepatic glucose output at the hyperinsulinemic state was increased in AD-CON. β-amyloid accumulation exacerbated hepatic insulin resistance, but all red pepper extract treatments reversed the insulin resistance in AD rats.

**Conclusions:**

The extracts of moderate and severe red peppers were found to prevent the memory deficit and exacerbation of insulin resistance by blocking tau phosphorylation and β-amyloid accumulation in diabetic rats with experimentally induced Alzheimer’s-like dementia. These results suggest that red pepper consumption might be an effective intervention for preventing age-related memory deficit.

## Background

Alzheimer’s dementia is a disease of slowly progressive synaptic collapse in the areas of the brain underlying memory and higher mental function [1-3]. As life expectancy increases worldwide, the incidence of dementia, especially Alzheimer’s disease, has also markedly increased, and the incidence of Alzheimer’s disease is expected to increase by about 3.1 folds from 2010 to 2050 [1,2]. However, the rapid increase may not be due to increased life expectancy alone. The increased incidence of Alzheimer’s disease may also be related to changes in lifestyles and diets such as lack of exercise and high fat and sugar diets although these possible causes remain controversial [4-8]. Recent studies have shown that obesity and high-fat diets increase the risk and/or progression of Alzheimer’s disease in humans [9,10], and diets high in fat also increase neuropathology and/or cognitive deficits in animal models of Alzheimer’s disease [6,7]. Ongoing evidence reveals that brain and possibly peripheral insulin resistance and concomitant hyperglycemia may be key metabolic dysfunctions contributing to Alzheimer’s disease [8,11,12]. Thus, it is important to identify dietary components that may prevent or delay both Alzheimer’s disease and diabetes.

Disturbances in insulin and possibly insulin growth factor-1 (IGF-1) signaling in the brain, especially the hippocampus, have been observed in Alzheimer’s disease. The deletion of insulin receptor and insulin receptor substrate (IRS)-2 in neurons delays brain growth and prevents memory dysfunction in mice by the ablation of the phosphoinositide-3-kinase/Akt/ mammalian target of rapamycin (mTOR) pathway in neurons [13]. The activation of mTOR suppresses the autophagic process in neurons and high concentrations of potentially toxic β-amyloid and tau proteins are sustained in the neurons [14]. However, by contrast, both IRS-1 and IRS-2 expressions decrease in neurons of humans with Alzheimer’s disease and both insulin and IGF-1 signaling are patently disturbed in brains affected by Alzheimer’s disease [15-17]. Type 2 diabetic patients have increased levels of hyperphosphorylated tau in their brains that facilitate the formation of neurofibrillary tangles that induce cognitive dysfunction [18]. Induction of experimental diabetes with streptozotocin or analogous drugs increases the levels of β-amyloid and tau phosphorylation in rodents [19,20]. Thus, the proper maintenance of insulin receptor signaling in the hippocampus plays an important role in cognitive function.

Due to the lack of effective treatments, it is necessary to develop preventive strategies to halt the development of Alzheimer’s disease in younger age people prior to onset of the disease. There is also a need to identify the environmental risk factors of Alzheimer’s disease. The non-genetic risk factors identified from cohort studies include caffeine consumption, lack of exercise, stress, less education, smoking, pesticide exposure and others [2,21].

Red pepper and its bioactive components such as capsaicinoids, capsinoids, and carotenoids are reported to decrease body fat mass by promoting fat oxidation in rats and humans [22,23]. Capsaicinoids, the substances responsible for the hot pungency of peppers, are known to induce pain by stimulating the transient receptor potential vanilloid 1 (TRPV1), but it also paradoxically desensitizes TRPV1 resulting in its common use in topical analgesic medications [24]. Although capsaicinoid desensitization of TRPV1 is mostly used topically, it is also known act systemically. Both pharmacological inhibition and genetic knockout of TRPV1 has been shown to prevent bone loss in ovariectomized mice [25]. Capsaicinoids are also known to consistently improve glycemic control, although their effects on insulin secretion have been controversial [23,25]. However, since impaired glucose tolerance and Alzheimer’s disease have a common etiological pathway, red pepper and its components might be beneficial for protecting against Alzheimer’s diseases. There are various types of red peppers according to the different combinations of bioactive components (capsaicin, capsaicinoids and flavonoids) which give the peppers various colors and pungencies [26]. Furthermore, red peppers with different colors and pungencies differently modulate energy and glucose homeostasis in OVX rats fed high fat diets [27]. Therefore, we hypothesized that specific varieties of red peppers might have a beneficial effect on preventing cognitive dysfunction and insulin resistance in type 2 diabetic rats with experimentally induced Alzheimer’s-type dementia. The objective of this study was to test the hypothesis using three varieties of red pepper from Young Yang County (Gungsangbuk-Do, Korea) according to the intensity of pungency: mild pungency red pepper (Geumdang, 1500 Scoville heat units), moderate pungency red pepper (Chilsung, 4000 Scoville heat units) and severe pungency red pepper (Subicho, 10,000 Scoville heat units) in partially pancretectomized rats, and also to explore possible mechanisms.

## Materials and methods

### Extraction and lyophilization

Three varieties of red pepper powders (mild, moderate and severe pungencies) were extracted with 70% ethanol by shaking for 24 h at 25°C, centrifuged at 8,000 × *g* for 30 min, and its supernatants were collected and supernatants lyophilized in a freeze-drier (Il Shin, Dongdochun-Si, Korea). Extraction using 70% ethanol is a very common extraction method for herbal preparations [28]. This method allows for the efficient extraction of many polar and non-polar compounds and is an efficient method for concentrating a wide variety of bioactive compounds including capsacinoids [28], bioflavonoids and carotenoids and without risk of toxic residue.

### Animals and diets

Male Sprague Dawley rats, weighing 192 ± 30 g, were housed individually in stainless steel cages in a controlled environment (23°C and a 12 hour light/dark cycle). All experimental procedures were performed according to the guidelines and with the approval of the Animal Care and Use Review Committee at Hoseo University, Korea. The rats had a 90% pancreatectomy (Px) using the Hosokawa technique and Px rats included in the experiments showed characteristics of type 2 diabetes after 2 weeks post-surgery [29]. Since the pancreas partially regenerates (to about 40% of the original volume) this technique results in a diminished insulin secretory capacity and inability to compensate for insulin resistance as typically seen in the Asian population.

After 7 days post Px surgery, rats were anesthetized with an intraperitoneal injection of a ketamine and xylazine mixture (100 mg and 10 mg/kg body weight, respectively) and placed in a stereotaxic device. A stainless steel cannula was implanted to stereotaxically connect an osmotic pump to the cannula implanted into the bilateral CA1 subregion using the following coordinates: lateral, −3.3 mm from the bregma; posterior, 2.0 mm from the midline; ventral, −2.5 mm from dura [30]. The β-amyloid (25–35) for AD and (35–25) for Non-AD were dissolved in sterile saline and infused into the cannula secured in bilateral CA1 subregions of the hippocampus using an osmotic pump (Alzet Osmotic Pump Company, Cupertino, CA, USA) at the rate of 3.6 nmol/day for 14 days. The β-amyloid (35–25) had the reverse sequence of β-amyloid (25–35) and does not accumulate in the brain and is not detected by immunohistochemistry (normal-control).

**Figure 1:**
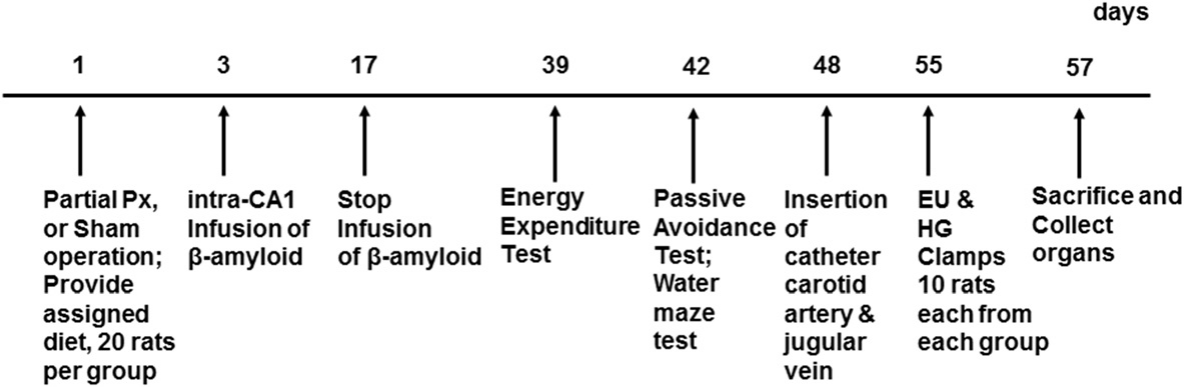
**Time table of the experimental studies.** EU clamp, euglycemic hyperinsulinemic clamp; HG clamp, hyperglycemic clamp.

Px rats infused with β-amyloid (25–35) were randomly assigned to four different groups of 20 animals each fed Western diets with elevated dietary fat to induce insulin resistance and supplemented with either 1% dextrin (AD-CON) or 1% of either mild pungency red pepper extract (AD-LP), moderate pungency red pepper extract (AD-MP), or sever pungency red pepper (AD-SP). Px rats infused with β-amyloid (35–25) had Western diets containing 1% dextrin as a normal control (Non-AD-CON). The dosage of red pepper extracts used in the present study is equivalent to approximately 3 g/day in humans and was based on extrapolation from our previous capsaicin and capsiate studies [27,23]. All rats freely consumed water and corresponding diets based on AIN-93 diet [31] for 47 days. All diets consisted of approximately 40 energy percent (En%) carbohydrates, 20 En% protein, and 40 En% fats. The major carbohydrate, protein and fat sources were starch plus sugar, casein (milk protein), and lard (CJ Co., Seoul). Figure 1 shows a time table of the experiments in the present study.

Serum glucose levels, food intakes, and body weights were measured weekly after overnight feed-deprivation. Homeostasis model assessment for insulin resistance index (HOMA-IR) was calculated as serum insulin levels (μU) × serum glucose levels (mM)/22.5.

### Energy expenditure by indirect calorimetry

After 39 days of the assigned treatment, energy expenditure was assessed at the beginning of the dark phase of the light–dark cycle after 6 h of feed-deprivation. The rats were placed into metabolic chambers (airflow = 800 ml/min) equipped with a computer-controlled O_2_ and CO_2_ measurement system (Biopac Systems Inc., Goleta, CA). The respiratory quotient (RQ) and resting energy expenditure (REE) were calculated using the equations provided by Niwa *et al.* [32]. Average oxygen consumption (VO_2_) and average carbon dioxide production (VCO_2_) were calculated using previously published methods and used to calculate carbohydrate and fat oxidation and the amount of oxygen consumed per gram of substrate oxidized [27].

### Locomotive activity

Locomotive activity was determined using a Linton AM1053 Activity Monitor consisting of a three-dimensional array of infrared beams placed around clear Perspex cages with AmLogger software (Linton Instruments, UK). The sum of rearing, mobility and activity measurements was used as a measure of total locomotive activity. Activity was assessed for 1 h during the dark phase of the light/dark cycle when the rats were most active after allowing 30 min to adapt to a clear Perspex cage.

### Passive avoidance test

On day 42 of the study, the rats were tested for short-term memory deficits using a passive avoidance apparatus consisting of a two-compartment dark/light shuttle box [30]. In the acquisition trial, electroshocks (75 V, 0.2 mA, 50 Hz) were delivered for 5 s, immediately after a rat had entered the dark chamber. Five seconds later, the rat was removed from the dark chamber and returned to its home cage. After 24 h, the retention latency time was measured in the same way as in the acquisition trial but electric foot shock was not delivered and the latency time was recorded to a maximum of 600 s. Short latencies indicate memory deficit, compared to significantly longer latencies.

### Water maze test

Spatial memory function was assessed with a Morris water maze test, as previously described [30], during day 44. The Morris water maze tests hippocampal-dependent learning, including the acquisition of spatial memory, long-term memory, and long-term spatial memory.

### Euglycemic hyperinsulinemic clamp

After catheterisation of the right carotid artery and left jugular vein in the 7th week, 10 rats were randomly selected and they were subjected to a euglycemic hyperinsulinemic clamp in a fasted conscious state (n = 10) to determine insulin resistance as previously described [23,33]. [3-^3^H] glucose (Perkin Elmer, Wellesley, MA) was continuously infused during a four-hour period at the rate of 0.05 μCi/min. Basal hepatic glucose output was measured in blood collected at 100 and 120 minutes after initiation of the (3-^3^H) glucose infusion. A primed continuous infusion of human regular insulin (Humulin; Eli Lilly, Indianapolis, IN) was then initiated at a rate of 20 pmol/kg bw/min to raise plasma insulin concentration to approximately 1100 pM after 210–240 min. Blood samples were collected at 10-minute intervals and 25% glucose was infused as needed to clamp glucose levels at approximately 6 mM. Rates of whole body glucose uptake and basal glucose turnover were determined according to the ratio of the [3-^3^H] glucose infusion rate to the specific activity of plasma glucose (dpm/mmol) during the final 30 minutes. Hepatic glucose production at the hyperinsulinemic clamped state was determined by subtracting the glucose infusion rate from the whole body glucose uptake.

### Hyperglycemic clamp

After seven weeks of treatment, catheters were implanted into the right carotid artery and left jugular vein of the remaining ten rats from each group as previously described [23,34]. At 5–6 days after implantation, a hyperglycemic clamp to determine insulin secretory capacity was performed in free-moving and overnight fasted rats. During the clamp, glucose infusion maintained serum glucose levels at 5.5 mM above the baseline and serum insulin levels were measured at 0, 2, 5, 10, 60, 90 and 120 min. Two days after the clamp, food-deprived rats were injected with human regular insulin (5 U/kg body weight) through the inferior vena cava. Ten min later, they were killed by decapitation, tissues collected and frozen in liquid nitrogen, and stored at −70°C. Liver glycogen content was determined after centrifuging lysates at 3000 rpm for 10 minutes and deproteinizing the supernatants with 1.5 N perchloric acid. The glycogen content was calculated from glucose released from glycogen hydrolyzed by α-amyloglucosidase in an acid buffer [27,35]. Serum insulin levels were measured using a rat ELISA kit (Linco). Triglyceride was extracted with chloroform-methanol (2:1, vol/vol) from the liver and resuspended in pure chloroform and determined as previously described [27,35] using a Trinder kit (Young Dong Pharm., Seoul, Korea).

### Brain and islet immunohistochemistry

The day after hyperglycemic clamp, overnight-fasted five rats were randomly selected from 10 rats of each group and were injected with BrdU (100 μg/kg body weight). Six hours post-injection, rats were anesthetized with intraperitoneal injections of a mixture of ketamine and xylazine, and the brain and pancreas was immediately dissected, perfused with saline and a 4% paraformaldehyde solution (pH 7.2) sequentially and postfixed with the same fixative overnight at room temperature [23,30].

Cryoprotected brain frozen tissues were serially sectioned on a cryostat (Leica, Wetzlar, Germany) into 30 μm coronal sections and β-amyloid accumulation in the hippocampus was determined by immunohistochemistry using β-amyloid antibody [30]. The β-amyloid deposition was calculated as the % β-amyloid-positive cells in the hippocampus area When sectioning paraffin-embedded pancreatic tissues, two of the 5-μm sections were discarded between each saved section to avoid duplicate analysis of the same islets when measuring the β-cell area, BrdU incorporation, and apoptosis were assessed as described previously using an immunohistochemistry method [23]. Pancreatic β-cell mass was estimated by multiplying the percentage of insulin-positive area by the weight of pancreatic tissues. The individual β-cell size was determined as the insulin-positive area divided by the number of nuclei counted in the corresponding insulin-positive structures in randomly immunofluoresence-stained sections using insulin antibody. BrdU incorporation in β-cells was determined with anti-insulin and anti-BrdU antibodies (R&D System, Minneapolis, MN) and β-cell proliferation was calculated as the total BrdU^+^ nuclei in β-cell nuclei per pancreas section. Apoptosis of β-cells was measured using a TUNEL kit (Roche Molecular Biochemicals, Indianapolis, IN) and counterstained with hematoxylin and eosin to visualize islets and apoptotic β-cells were measured by the total number of apoptotic bodies in β-cell nuclei per pancreas section.

### Immunoblot analysis

The day after hyperglycemic clamp, four overnight-fasted rats were randomly selected from 10 rats of each group and the hippocampi from the four rats were isolated. They were lysed in 20 mM Tris buffer (pH 7.4) containing 2 mM EDTA, 137 mM NaCl, 1% NP40, 10% glycerol and 12 mM α-glycerol phosphate and protease inhibitors. After 30 min on ice, the lysates were centrifuged for 10 min at 12,000 rpm at 4°C. After measuring protein contents in lysates using a Bio-Rad protein assay kit, lysates with equal amounts of protein were immunoprecipitated with specific antibodies prior to separation by SDS-PAGE as previously described [23,30]. Antibodies used for the immunoblot analysis were cAMP responding element binding protein (CREB), phosphorylated CREB^ser133^, protein kinase B (PKB or Akt), phosphorylated PKB^Ser473^, glycogen synthase kinase (GSK)-3β, phosphorylated GSK-3β^ser9^, tau, phophorylated tau^ser396^ and β-actin (Cell Signaling Technology). The intensity of protein expression was determined using Imagequant TL (Amersham Biosciences).

### Statistical analysis

All results were expressed as a mean ± SD. Statistical analyses were performed using SAS version 7 (SAS Institute). All variables exhibited normal distribution in univariate analysis. One-way ANOVA was used to determine animal group effects separately for each time point and each treatment. Differences among groups with a P < 0.05 were considered statistically significant by Tukey’s test.

## Results

### Hippocampal β-amyloid deposition and cognitive function

**Figure 2:**
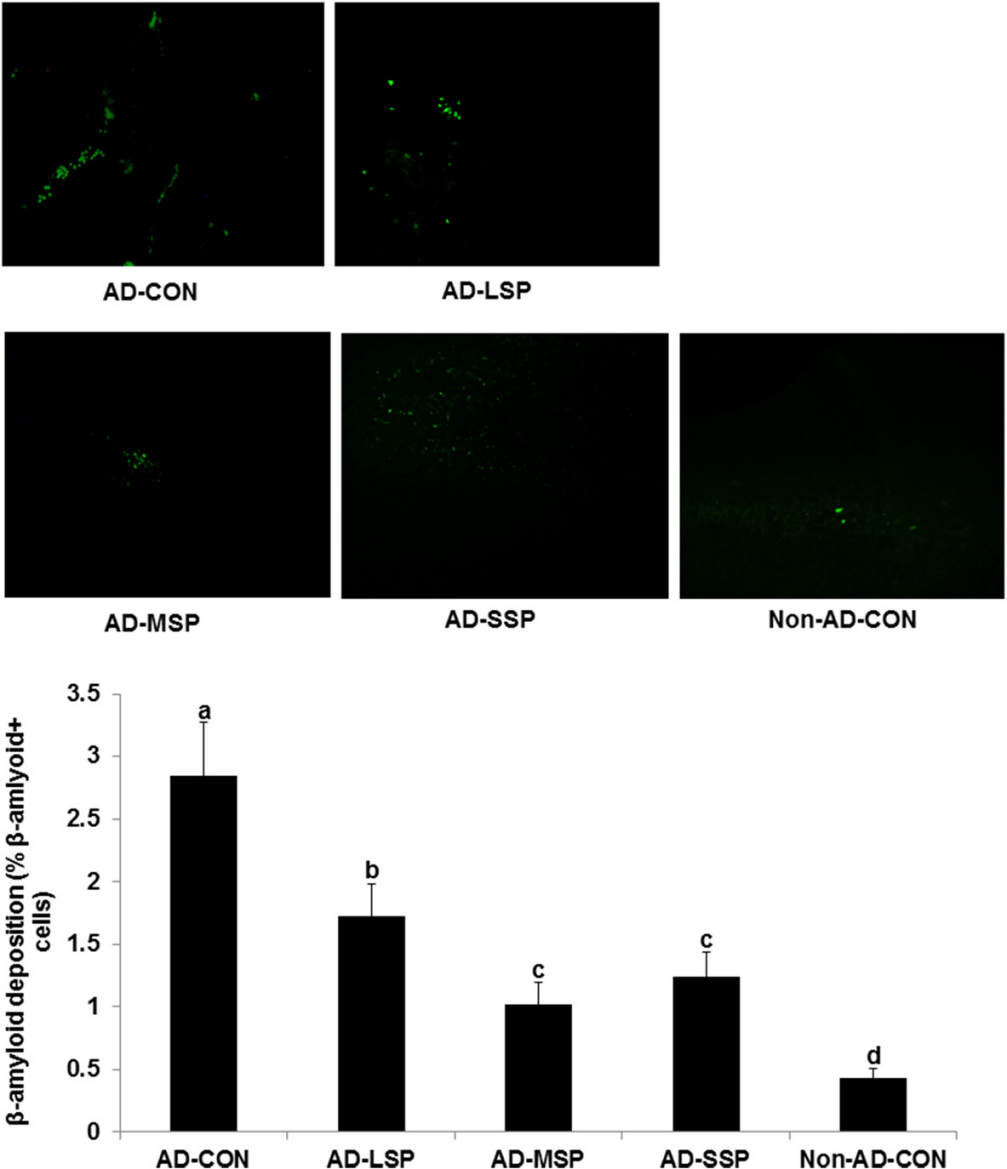
**β-amyloid staining in the hippocampus.** At the end of experimental period, the brain section (30 μm) was stained for β-amyloid using immunohistochemical techniques. The β-amyloid positive cells were counted in the hippocampus area. The β-amyloid (25–35) infused diabetic rats fed high fat diets with 1% dextrin (AD-CON), 1% less pungent red pepper (AD-LSP), moderate pungent red pepper (AD-MSP) or severe pungent red pepper (AD-SSP) for 28 days. The β-amyloid (35–25) infused rats fed high fat diets with 1% dextrin (Non-AD-CON). Each bar represents Means ± SD (n = 5). ^abcd^Different letters on the bars indicate significant differences at P < 0.05.

The β-amyloid (25–35) immunoreactivities of the hippocampi were markedly greater in AD-CON compared to Non-AD-CON, and the immunostaining was decreased in descending order of LP < MP < SP < CON in the β-amyloid infused diabetic rats (Figure 2).

**Figure 3:**
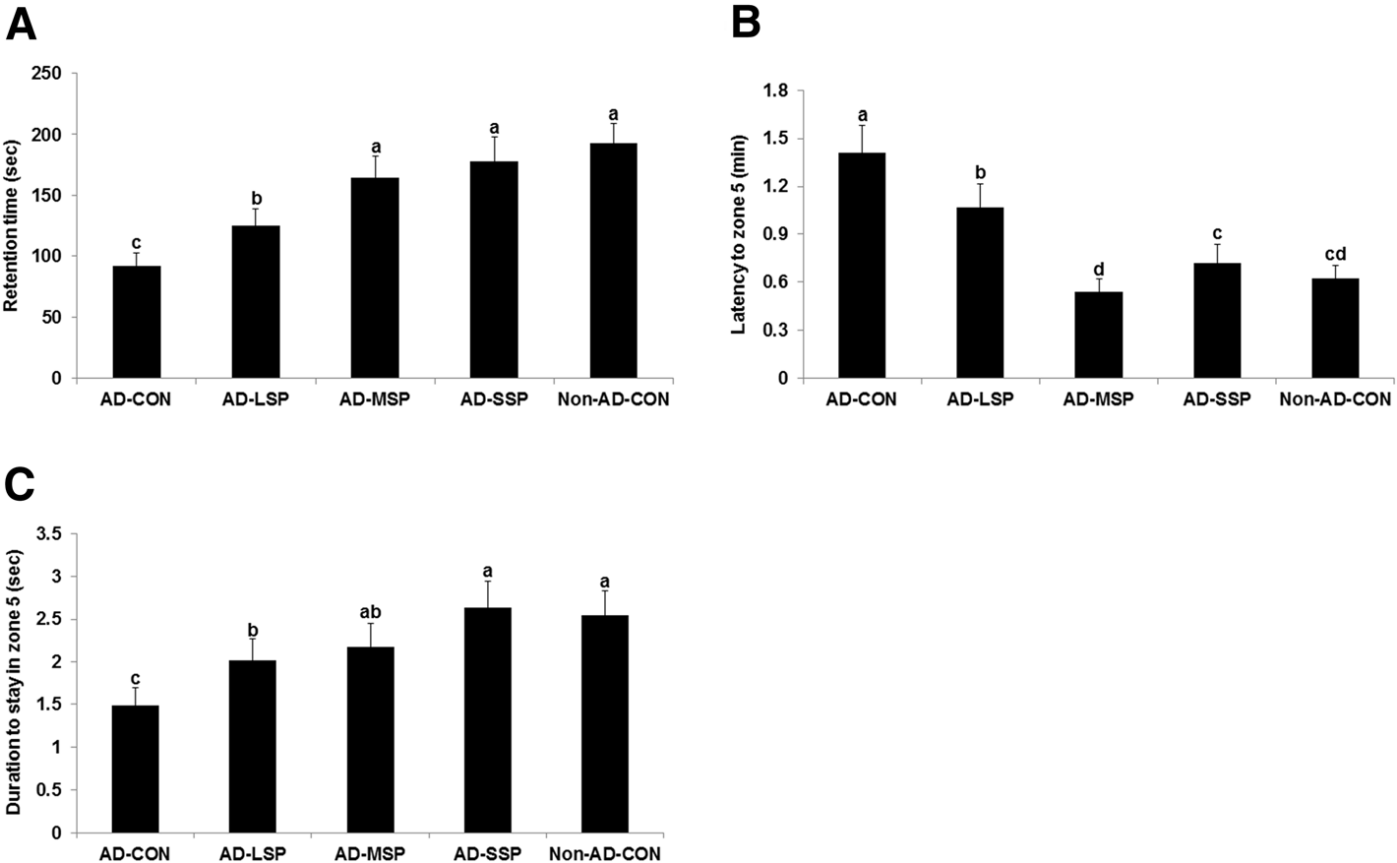
**Memory, cognitive function and behavior changes of rats with β-amyloid infusion.** Latency time to enter the dark room in passive avoidance test **(A)** and the latency to locate zone with the platform during **(B)** and the period to stay in the platform zone on day 5 during water maze test **(C)** were given. The β-amyloid (25–35) infused diabetic rats fed high fat diets with 1% dextrin (AD-CON), 1% less pungent red pepper (AD-LSP), moderate pungent red pepper (AD-MSP) or severe pungent red pepper (AD-SSP) for 28 days. The β-amyloid (35–25) infused rats fed high fat diets with 1% dextrin (Non-AD-CON). Each dot and bar represents Means ± SD (n = 20). ^abcd^Different letters on the bars indicate significant differences at P < 0.05.

In the passive avoidance test, AD-C had a shorter retention time to enter the lighted room than Non-AD-CON despite previous experience of electric shock upon entering the light room. This indicated that AD-C had impaired short-term memory in comparison to Non-AD-CON. The retention time was made longer with SP = MP < LP < CON in the β-amyloid infused diabetic rats (Figure 3A). In addition, the water maze test revealed that the rats of the Non-AD-CON group swam across the pool and quickly reached the platform, rapidly learning its location, but the rats of the AD-CON took a significantly longer retention time to locate the platform at zone 5 and were in zone five for a shorter time (Figure 3B). Rats in the LP < SP < MP groups found zone 5 locating the platform faster than AD-CON and rats in the AD-CON < LP < MP and SP stayed in zone 5 longer (Figure 3B). Thus, AD-CON infused with β-amyloid (25–35) experienced a decline in short term and spatial memory, which was fully or partially prevented by MP = SP > LP.

### Hippocampal insulin signaling

**Figure 4:**
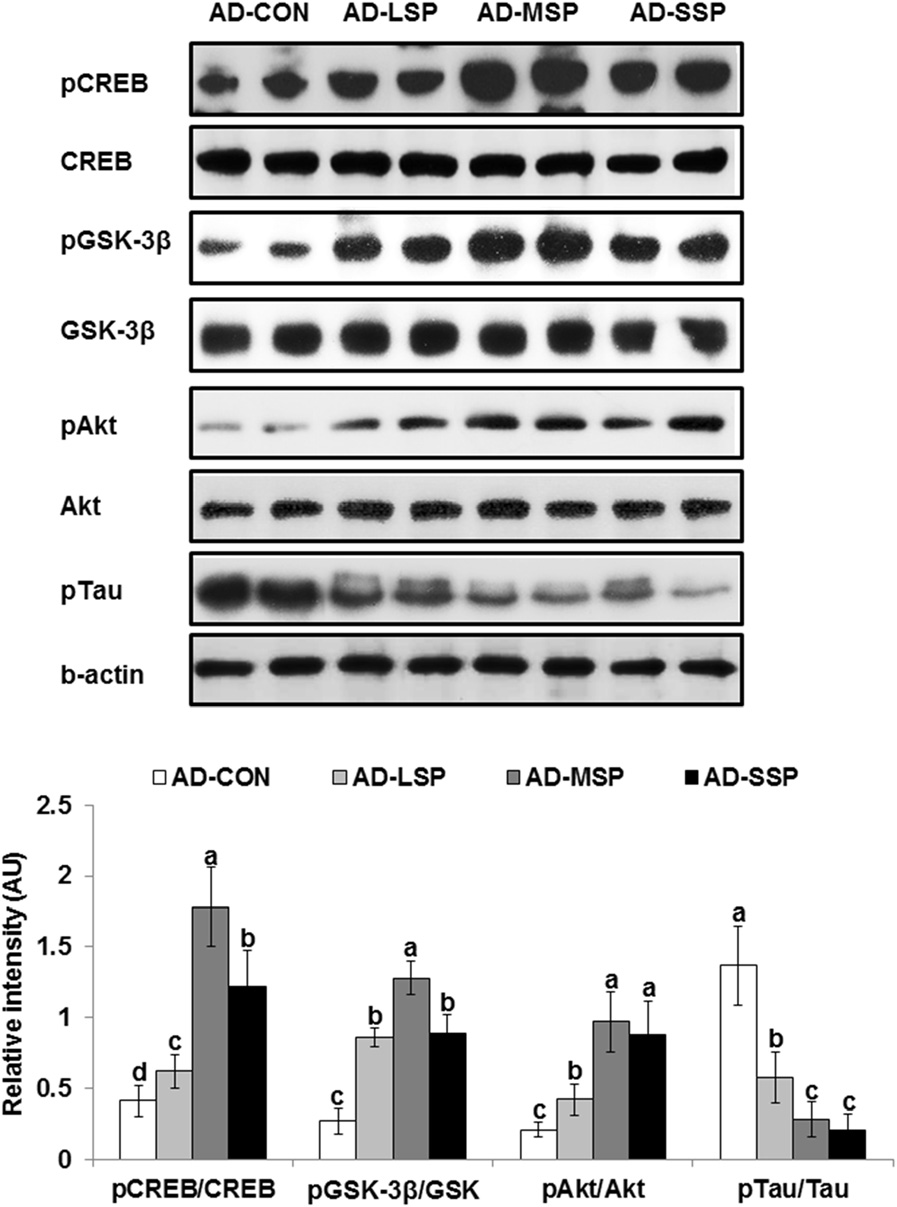
**Insulin signaling in the hippocampus.** The β-amyloid (25–35) infused diabetic rats fed high fat diets with 1% dextrin (AD-CON), 1% less pungent red pepper (AD-LSP), moderate pungent red pepper (AD-MSP) or severe pungent red pepper (AD-SSP) for 28 days. The β-amyloid (35–25) infused rats fed high fat diets with 1% dextrin (Non-AD-CON). CREB, cAMP responding element binding protein; pCREB, phosphorylated CREB; Akt, protein kinase B; pAKT, phosphorylated Akt; GSK, glycongen synthase kinase; pGSK, phosproylated GSK; Tau, tau protein; pTau, phosphorylated Tau protein. Each bar represents Means ± SD (n = 4). ^abcd^Different letters on the bars indicate significant differences at P < 0.05.

The phosphorylation of CREB increased with MP and SP in AD rats in comparison to the control group. As an indicator of insulin signaling, the phosphorylation of Akt was also higher in LP, MP, and SP than the control groups and the increment for MSP was highest among all groups (Figure 4). The phosphorylation of GSK-3β, a downstream of GSK, exhibited the same pattern of phosphorylation as Akt. The phosphorylation of tau was lower in descending order of control > LP > MP and SP. Therefore, MP and SP consumption prevented the attenuation of hippocampal insulin signaling and the prevention resulted in lower tau phosphorylation.

### Energy metabolism

In diabetic rats AD-CON tended to have lower body weight than Non-AD-CON, but it was not significantly different. Rats in the LP and SP groups did not change body weight in comparison to AD-CON but MP somewhat increased up to those of the Non-AD-CON group. However, epididymal and retroperitoneal fat mass relative to body weight were significantly higher in the AD-CON group than the Non-AD-CON group. LP, MP and SP supplementations all resulted in fat masses similar to the Non-AD-CON group (Table 1). Energy intake tended to be lower in the AD-CON compared to the Non-AD-CON rats, but LP, MP and SP prevented its decrease in AD rats. However, locomotive activity was decreased in AD-CON rats and MP and SP inhibited the decrease. Daily energy expenditure was not different between AD-CON and Non-AD-CON and oxygen consumption and respiratory quotient were not different among the groups. However, SP increased oxygen consumption and daily energy expenditure in comparison to AD-CON but respiratory quotient was not significantly different among the groups. Carbohydrate oxidation was greater and fat oxidation lower in the AD-CON group compared to the Non-AD-CON. MP and SP reversed these differences in energy nutrient oxidation in AD rats, and AD rats given MP and SP had greater fat oxidation more than Non-AD-CON rats.

**Table 1:** **Body weight visceral fat contents and overnight-fasted serum glucose and insulin levels at the end of experimental periods**

	**AD-CON (n = 20)**	**AD-LSP (n = 20)**	**AD-MSP (n = 20)**	**AD-SSP (n = 20)**	**Non-AD-CON (n = 20)**
Body weight (g)	320 ± 33^b^	317 ± 29^b^	333 ± 34^ab^	322 ± 30^b^	348 ± 31^a^
Epididymal fat pads/body weight (g/kg bw)	9.0 ± 1.2^a^	7.1 ± 0.9^b^	7.4 ± 1.0^b^	7.2 ± 0.9^b^	7.5 ± 1.0^b^
Retroperitoneal fat/body weight (g/kg bw)	15.5 ± 2.0^a^	12.4 ± 1.9^b^	13.2 ± 1.9^b^	12.5 ± 1.8^b^	12.9 ± 1.8^b^
Energy intake (kcal/day)	105 ± 17	114 ± 15	119 ± 21	118 ± 19	113 ± 14
Total active time (m/h)	4.4 ± 0.7^b^	5.2 ± 0.8^ab^	5.7 ± 0.8^a^	5.4 ± 0.6^a^	5.5 ± 0.6^a^
Energy expenditure (kcal/kg^0.75^/day)	119 ± 15^b^	126 ± 15^ab^	129 ± 16^ab^	133 ± 16^a^	111 ± 14^b^
Respiratory quotient	0.86 ± 0.11	0.84 ± 0.10	0.81 ± 0.10	0.80 ± 0.09	0.83 ± 0.10
VO_2_ (mL/kg^0.75^/min)	17.0 ± 2.1^b^	17.9 ± 2.2^ab^	18.4 ± 2.2^ab^	19.0 ± 2.1^a^	15.9 ± 2.0^b^
Carbohydrate oxidation (mg/kg^0.75^/min)	6.6 ± 0.9^a^	6.1 ± 0.7^ab^	4.8 ± 0.6^b^	5.0 ± 0.6^b^	4.8 ± 0.6^b^
Fat oxidation (mg/kg^0.75^/min)	6.1 ± 0.8^c^	7.3 ± 0.9^b^	8.9 ± 1.1^a^	9.2 ± 1.1^a^	7.1 ± 0.9^b^

Values are mean ± SD. The β-amyloid (25–35) infused diabetic rats fed high fat diets with 1% dextrin (AD-CON), 1% less pungent red pepper (AD-LSP), moderately pungent red pepper (AD-MSP) or severely pungent red pepper (AD-SSP) for 28 days. The β-amyloid (35–25) infused rats fed high fat diets with 1% dextrin (Non-AD-CON).

^abc^Different superscript alphabets on the same row indicate significant differences at P < 0.05.

### Insulin resistance

**Figure 5:**
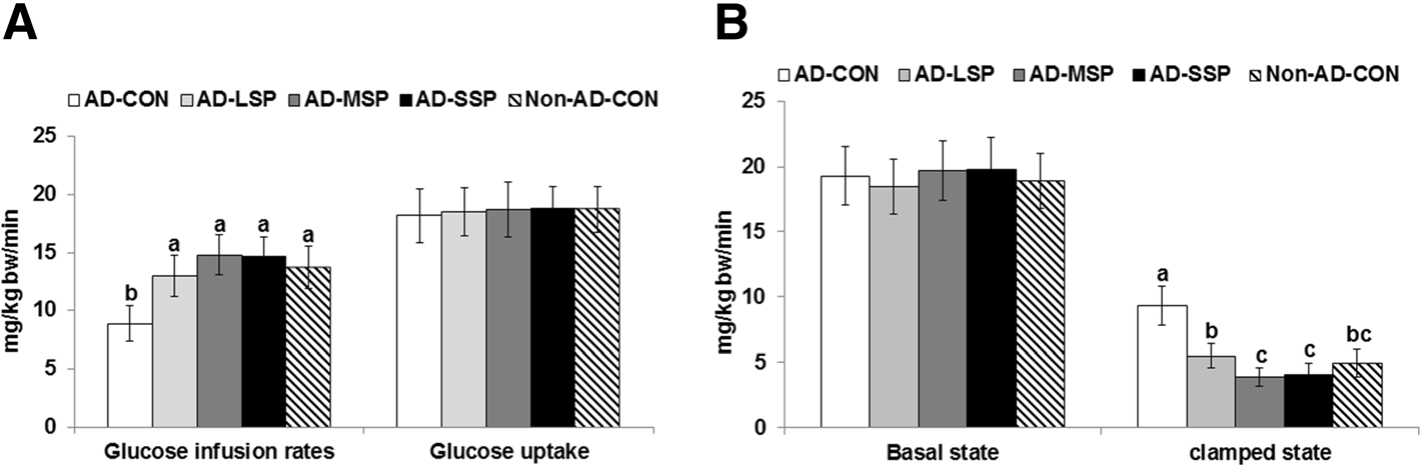
**Glucose infusion rates and hepatic glucose output during a euglycemic hyperinsulinemic clamp.** Euglycemic hyperinsulinemic clamp was performed in conscious, free moving, and overnight fasted rats to determine whole body insulin resistance. Glucose infusion rates and glucose uptake at a clamped steady-state **(A)** and hepatic glucose output at baseline and hyperinsulinemic state about 1100 pM of serum insulin **(B)** were presented. The β-amyloid (25–35) infused diabetic rats fed high fat diets with 1% dextrin (AD-CON), 1% less pungent red pepper (AD-LSP), moderate pungent red pepper (AD-MSP) or severe pungent red pepper (AD-SSP) for 28 days. The β-amyloid (35–25) infused rats fed high fat diets with 1% dextrin (Non-AD-CON). Each bar represents Means ± SD (n = 10). ^abc^Different letters on the bars indicate significant differences at P < 0.05.

In comparison with Non-AD-CON rats, AD-CON rats displayed decreased glucose infusion rates by about 29% at hyperinsulinemic clamped states (about 1100 pM serum insulin levels). However, the decrease in AD rats was prevented by LP, MP and SP treatments (Figure 5A). Hepatic glucose output in the basal state was not significantly different between AD-CON and Non-AD-CON rats and none of the treatments altered it in AD rats (Figure 5B). However, hepatic glucose output at the hyperinsulinemic state was higher in AD-CON than Non-AD-CON, but it was suppressed by LP < MP = SP in AD-rats. Since glucose uptake was not significantly different among the groups, a good portion of the difference in glucose infusion rates could be accounted for by the insulin-stimulated decreases in hepatic glucose production. This indicated that the exacerbation of insulin resistance in the AD-CON group was mainly associated with hepatic insulin resistance.

### Insulin secretion capacity

Serum glucose levels in the overnight fasted state did not differ between AD-CON and Non-AD-CON rats and the treatment with red pepper extracts did not alter the levels in AD rats. However, overnight fasted serum insulin levels were higher in the AD-CON group than Non-AD-CON whereas they were lower in rats fed LP, MP and SP. When calculating insulin resistance from serum glucose and insulin levels at the fasted state, it was higher in the AD-CON group than the Non-AD-CON group and all of the treatments lowered it to similar levels as Non-AD-CON (Table 1).

**Figure 6:**
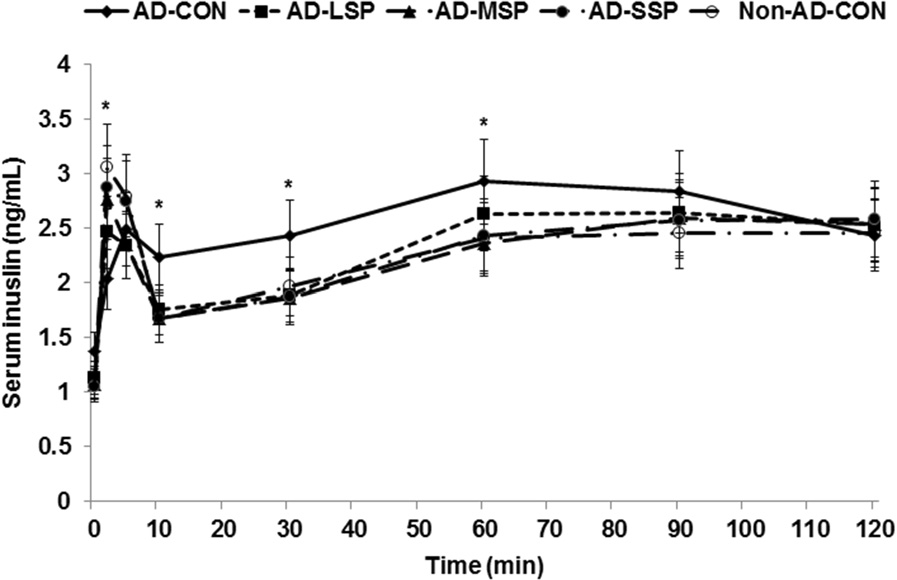
**Insulin secretion during a hyperglycemic clamp.** Hyperglycemic clamp was conducted in conscious, free moving, and overnight fasted rats to measure glucose-stimulated insulin secretion. As exogenous glucose was infused into jugular vein to make approximately 5.5 mM above overnight fasted serum glucose levels, serum insulin levels were measured at 0, 2, 5, 10, 30, 60, 90 and 120 mins. The β-amyloid (25–35) infused diabetic rats fed high fat diets with 1% dextrin (AD-CON), 1% less pungent red pepper (AD-LSP), moderate pungent red pepper (AD-MSP) or severe pungent red pepper (AD-SSP) for 28 days. The β-amyloid (35–25) infused rats fed high fat diets with 1% dextrin (Non-AD-CON). Each dot represents Means ± SD (n = 10). *Significantly different among the groups at P < 0.05.

To confirm the above observations, hyperglycemic clamp was performed on rats in all groups. During hyperglycemic clamp was used to determine β-cell function, insulin secretion was biphasic, with a first and second phase. In the first phase, plasma insulin levels peaked between 2 and 5 min in an acute state and then declined to a nadir at 10 min. An ascending second phase of plasma insulin was observed at 60 min and it was sustained until 90–120 min (Figure 6). Average and AUC of serum insulin levels were higher in AD-CON rats in the first and second phases of insulin secretion than those of Non-AD-CON rats (Table 2). LP, MP and SP treatments resulted in similar serum insulin levels at the first phase as the AD-CON group (Table 2). However, all of treatments inhibited the increase in insulin secretion at the second phase. Thus, this suggests that LP, MP and SP prevented rampant insulin secretion during the second phase in AD rats without suppressing the first phase insulin secretion.

**Table 2:** **Glucose homeostasis and insulin secretion during hyperglycemic clamp**

	**AD-CON (n = 20)**	**AD-LSP (n = 20)**	**AD-MSP (n = 20)**	**AD-SSP (n = 20)**	**Non-AD-CON (n = 20)**
Serum glucose at basal state (mM)	7.1 ± 0.8	6.9 ± 0.7	7.1 ± 0.7	7.0 ± 1.0	7.0 ± 0.6
Serum insulin (ng/mL)	1.37 ± 0.17^a^	1.13 ± 0.15^b^	1.07 ± 0.16^b^	1.05 ± 0.14^b^	1.09 ± 0.15^b^
HOMA-IR	12.6 ± 2.5^a^	9.4 ± 2.1^b^	9.1 ± 1.9^b^	8.8 ± 1.9^b^	9.2 ± 2.1^b^
First phase of insulin (0–10 min; ng/mL)	2.25 ± 0.35	2.18 ± 0.32	2.26 ± 0.33	2.44 ± 0.34	2.51 ± 0.37
Second phase of insulin (60–120 min; ng/mL)	2.66 ± 0.38^a^	2.42 ± 0.40^ab^	2.34 ± 0.34^b^	2.36 ± 0.32^b^	2.32 ± 0.34^b^
AUC of first phase of insulin (0–10 min; ng/mL*min)	26.3 ± 3.8	25.8 ± 3.5	26.7 ± 3.6	29.1 ± 3.6	30.0 ± 4.2
AUC of second phase of insulin (30–120 min; ng/mL*min)	246 ± 33^a^	225 ± 31^ab^	215 ± 29^b^	217 ± 27^b^	212 ± 28^b^
Glucose infusion rate (mg/kg bw/min)	8.6 ± 1.5^c^	11.6 ± 1.8^b^	12.8 ± 2.0^ab^	13.2 ± 2.1^a^	13.4 ± 1.9^a^
Insulin sensitivity at hyperglycemic state (μmol glucose · min^−1^ · 100 g^−1^ per μmol insulin/L)	3.2 ± 0.6^c^	4.8 ± 0.7^b^	5.5 ± 0.8^a^	5.6 ± 0.8^a^	5.8 ± 0.8^a^

Values are Means ± SD. Values are mean ± SD. The β-amyloid (25–35) infused diabetic rats fed high fat diets with 1% dextrin (AD-CON), 1% less pungent red pepper (AD-LSP), moderately pungent red pepper (AD-MSP) or severely pungent red pepper (AD-SSP) for 28 days. The β-amyloid (35–25) infused rats fed high fat diets with 1% dextrin (Non-AD-CON). HOMA-IR, homeostatic model assessment for insulin resistance. First phase of serum insulin was defined as the average of serum insulin levels at 2 and 5 mins, with second phase at 60, 90 and 120 mins. Insulin sensitivity at hyperglycemic state was calculated as the ratio of glucose infusion rate to steady-state plasma insulin levels.

^abc^Different superscript alphabets on the same row indicate significant differences at P < 0.05.

Glucose infusion rates in hyperglycemic clamp indicated β-cell function and insulin sensitivity at the hyperglycemic state, calculated as the ratio of glucose infusion rates to steady-state serum insulin levels [23,33]. Glucose infusion rates required to maintain hyperglycemia at 5.5 mM above baseline were found to be greater in the descending order of the MP = SP < LP = control groups (Table 2). Insulin sensitivity at the hyperglycemic state was lower in AD-CON than Non-AD-CON whereas it was enhanced in the ascending order of control < LP = MP < SP in AD-CON rats. SP showed a comparable insulin sensitivity to Non-AD-CON at the hyperglycemic state (Table 2). Therefore, AD-CON exhibited disrupted regulation of insulin secretion and attenuated insulin sensitivity at the hyperglycemic state in comparison to the Non-AD-CON, but LP, MP and SP prevented the disruption of insulin secretion and insulin resistance and was similar to Non-AD-CON.

### Pancreatic β-cell mass, proliferation and apoptosis

Pancreatic β-cell area was estimated from the combination of the number and size of β-cells. The percentage of β-cell area was significantly lower in AD-CON rats than Non-AD-CON rats, whereas LP treatment in AD rats increased the area (Table 3). Individual β-cell size, indicating β-cell hypertrophy, was higher in AD-CON rats than in Non-AD-CON rats and LP and MP in AD rats lowered them to a similar size as the Non-AD-CON size. However, pancreatic β-cell mass, calculated by multiplying β-cell area by the pancreas weight was significantly lower in the AD-CON than the Non-AD-CON group, whereas LP and MP increased total β-cell mass significantly, to as much as the Non-AD-CON (Table 3). Islet morphometry revealed that proliferation of β-cells was not significantly different between AD-CON and Non-AD-CON groups and none of the treatments with red pepper extracts altered β-cell proliferation (Table 3). In contrast, AD-CON rats had elevated β-cell apoptosis compared to Non-AD-CON rats and the elevation was suppressed by LP, MP and SP treatments (Table 3).

**Table 3:** **Islet morphometry**

	**AD-CON (n = 5)**	**AD-LSP (n = 5)**	**AD-MSP (n = 5)**	**AD-SSP (n = 5)**	**Non-AD-CON (n = 5)**
β-cell area (%)	5.3 ± 1.1^b^	6.4 ± 0.9^b^	5.9 ± 0.8^ab^	5.7 ± 1.0^ab^	7.9 ± 1.0^a^
Individual β-cell size (μm^2^)	261 ± 30^a^	228 ± 29^b^	235 ± 28^b^	241 ± 27^ab^	234 ± 27^b^
Absolute β-cell mass (mg)	21.0 ± 3.1^b^	24.7 ± 2.9^a^	24.0 ± 2.9^a^	23.5 ± 2.8^ab^	24.9 ± 2.9^a^
BrdU^+^ cells (% BrdU^+^ cells of islets)	0.94 ± 0.13	1.03 ± 0.12	1.05 ± 0.11	1.06 ± 0.12	1.01 ± 0.14
Apoptosis (% apoptotic bodies of islets)	1.19 ± 0.16^a^	0.97 ± 0.12^b^	1.02 ± 0.12^b^	1.02 ± 0.13^b^	0.95 ± 0.13^b^

Values are Means ± SD. The β-amyloid (25–35) infused diabetic rats fed high fat diets with 1% dextrin (AD-CON), 1% less pungent red pepper (AD-LSP), moderately pungent red pepper (AD-MSP) or severely pungent red pepper (AD-SSP) for 28 days. The β-amyloid (35–25) infused rats fed high fat diets with 1% dextrin (Non-AD-CON).

^ab^Different superscript alphabets on the same row indicate significant differences at P < 0.05.

## Discussion

Red pepper is known to prevent obesity by increasing energy expenditure and improving glucose homeostasis [26,27]. Furthermore, red pepper types with different pungencies have been shown to differently affect energy and glucose homeostasis in ovariectomized rats [27]. This is related to the contents of capsaicinoids, total flavonoids and total phenolic compounds. Our previous study revealed that the contents of total capsaicinoids were higher in the descending order of SP, MP and LP whereas those of total flavonoids and total phenolic compounds were higher in ascending order of SP, LP and MP [27]. The present study showed that the efficacies of the extracts were: control < LP < MP and SP for preventing memory deficit and for decreasing β-amyloid accumulation in the hippocampus through potentiating pCREB➔pGSK and lowering pTau. In addition, the red pepper extracts improved hepatic insulin sensitivity in rats with Alzheimer’s like symptoms. Insulin secretion especially as measured in the second phase was greater in AD rats than Non-AD rats and LP, MP and SP lowered it. Β-cell mass was lower in AD rats due to increased β-cell apoptosis than Non-AD rats and all red pepper extracts prevented the decrease. These results demonstrate that MSP and SSP can prevent both the cognitive dysfunction and hepatic insulin resistance exhibited by this animal model of type 2 diabetic rats with experimentally induced AD.

The pathogenesis of Alzheimer’s disease is not yet fully understood. However, the formation of β-amyloid plaques play a crucial a role in Alzheimer’s diseases in humans. β-amyloid infusion into the lateral ventricle or CA1 region in the hippocampus of animals also induces hyperphosphorylated tau aggregates, neurofibrillary tangles and concomitant cognitive dysfunction [30,36]. These animals exhibit similar characteristics to those of humans with Alzheimer’s diseases. The symptoms are associated with the hyperphosphorylation of tau, a key factor in the formation of neurofibrilary tangles [8,37]. The hyperphosphorylation is associated with brain insulin resistance that results from the impairment of several pathways such as insulin and IGF-1 signaling [18,37,38]. IGF-1 and insulin signaling are connected to CREB phosphorylation in the brain, especially in the hippocampus. The neurons express CREB, which is associated with memory function and CREB phosphorylation plays a critical step for the initiation of learning and memory-required gene transcription [39]. Since the activation of TRPV1 also potentiates the phosphorylation of CREB that increases the expression of IRS2 especially in the liver and brain [40], TRPV1 activation may enhance IGF-1 signaling. Furthermore, several lines of scientific evidence supports that TRPV1 is involved in cognitive function [41]. First of all, the TRPV1 is widely expressed in the hippocampus [42]. Second, the activation of TRPV1 increases the release of calcitonin gene-related peptide (CGRP) from sensory and dorsal root ganglion neurons dose-dependently [37,40]. CGRP is reported to increase IGF-1 production in the astrocytes in the hippocampus [43]. Thus, TRPV1 agonists such as capsaicin may prevent brain insulin resistance by the activation of insulin/IGF-1 signaling.

Red peppers contain many bioactive components such as capsaicin, dihydrocapsaicin, β-carotene, zeaxanthin, capsanthin, total carotenoids and chlorogenic acid [27]. The contents of capsaicin, dihydrocapsaicin and total capsaicinoids were greater in an ascending order of mildly pungent red pepper (Geumdang) < moderate (Chilsung) < severe (Subicho) in our previous study [25]. This is related to the amount of TRPV1 agonists with different pungency such as capsaicin (pungent) and capsiate (non-pungent) that have different activities for energy metabolism and glucose homeostasis in diabetic rats [23]. Capsaicin and capsiate have better activity for increasing energy metabolism and glucose tolerance, respectively [23]. In addition, severely pungent red pepper was shown to decrease fat mass and increase energy expenditure better than less severe pungent red pepper whereas less pungent red pepper was more effective for improving insulin sensitivity and hepatic insulin signaling [27]. Peppers differently affect energy balance and glucose homeostasis according to color and pungency due to their different bioactive components. The present study also found that pungency of red pepper determined the effectiveness for improving cognitive function: capsaicin-rich red peppers such as MP and SP improved cognitive function and potentiated hippocampal IGF-1 and insulin signaling (pCREB ➔ pAkt ➔ pGSk-3b) better that LP in the hippocampus. Capsaicin and dihydrocapsacin were apparently the major components that enhanced cognitive function by decreasing β-amyloid accumulation, but their contents in MSP may be sufficient to show maximum activity when consumed at 1% of the diet since MP and SP were equally effective for improving cognitive function. Thus, the components that activate TRPV1 may mitigate cognitive dysfunction through IGF-1 signaling in the hippocampus.

The deposition of β-amyloid in the brain induces brain and possibly peripheral insulin resistance that is a common pathophysiological feature of Alzheimer’s diseases and type 2 diabetes [11,44]. Alzheimer’s disease patients also have impaired glucose regulation but the dysregulation is not as severe as in type 2 diabetes [45], since glucose homeostasis is a result of both insulin resistance and insulin secretion. Type 2 diabetes is induced when insulin secretion cannot compensate for insulin resistance. However, many Alzheimer’s disease patients release sufficient insulin to compensate for insulin resistance and do not progress to type 2 diabetes [44]. The present study found that β-amyloid deposition increased in the control group and exacerbated peripheral insulin resistance whereas MP and SP had fewer β-amyloid tangles and better peripheral insulin sensitivity. Since capsaicin is known to activate TRPV1 [46], and MP and SP contained more capsaicin than LP, the former might activate the TRPV1 more than the latter. The activation of TRPV1 probably decreased β-amyloid plaques by inhibiting tau phosphorylation and it may be associated with decreasing peripheral insulin resistance. Since LSP improved glucose homeostasis better than MSP and SSP in ovariectomized rats fed a high fat diet in our previous study [27], the enhancement of glucose homeostasis by MP and SP in β-amyloid infused rats was due to the decrease in β-amyloid deposition in the hippocampus that exacerbated insulin resistance in the present study. Thus, these results suggested that the consumption of MP and SP resulted in the lower β-amyloid deposition in the hippocampus and the decrease prevented insulin resistance.

The brain plays an important role in regulating glucose homeostasis. The brain and peripheral tissues, especially the liver and islets, are connected and reciprocally deliver messages [47,48]. It is well-known that the hypothalamus is a crucial organ in the regulation energy and glucose metabolism. The hippocampus is a brain region where neurogenesis continues throughout life and its neuronal death results in altered neuronal circuits and impaired learning and memory [49]. Neurogenesis in the brain, especially the hippocampus, ameliorates cognitive dysfunction by insulin and Wnt signaling [48,49]. Therefore, neuronal loss in the hippocampus due to β-amyloid deposition might induce glucose dysregulation by exacerbating peripheral insulin resistance, especially in the liver. Furthermore, plasma β-amyloid (40/42) levels are increased in Alzheimer’s disease patients with hyperglycemia and they exacerbate hepatic insulin resistance by activating Janus kinase-2 signaling in the liver [50]. The present study showed that MP and SP prevented the pathway to cognitive dysfunction and hepatic insulin resistance in rats induced cognitive dysfunction by β-amyloid accumulation in the hippocampus.

In addition to peripheral insulin resistance, brain pathologies associated with AD affect insulin secretion by impeding the cross-talk between the brain and islets via the sympathetic nervous system and adipokines [30,44,51]. The accumulation of β-amyloid did not exacerbate the first phase of glucose tolerance possibly due to a slight increase in insulin secretion, but serum glucose levels during the second phase reached a higher peak and decreased slowly, indicating severe glucose intolerance and insulin resistance [30,44]. Hyperglycemic clamp demonstrated that the insulin secretion during 10–60 min was higher in β-amyloid infused rats, but the insulin secretion during 60–120 min decreased [44]. MP and SP protected against the higher glucose levels during 10–60 min and the insulin secretion of the second phase was maintained. These results indicate that hippocampal β-amyloid deposition associated with cognitive dysfunction might impair also the control of insulin secretion by disrupting the tight regulation of insulin secretion regardless of insulin resistance. β-cell function is related to β-cell mass [23,51]. When insulin secretion is not tightly regulated due to a certain condition such as insulin resistance, β-cell size is increased but the number of β-cells is not elevated [23]. As a result, β-cell mass is eventually decreased in an insulin resistant state. In addition, insulin secretory capacity is markedly decreased when β-cell mass is decreased due to increased β-cell apoptosis and/or lowered β-cell proliferation [23]. The present study demonstrated that β-cell mass was decreased resulting in a lower maximal insulin secretion due to increased β-cell apoptosis, but MSP and SSP protected against the impairment of β-cell function and loss of mass. However, mice lacking TRPV1 had a more youthful metabolic profile due at least in part to greater maximal insulin secretion due to greater β-cell mass as a consequence of less apoptosis [43]. These results appear contradictory to the present study, and are similar to our previous study with long-term consumption of capsaicin [23,27]. However, long-term capsaicin consumption may desensitize the TRPV1. Further studies need to determine whether long-term consumption of capsaicin activates or desensitizes the TRPV1.

## Conclusions

Hippocampal β-amyloid accumulation induced cognitive dysfunction and impaired glucose homeostasis by exacerbating insulin resistance and decreasing β-cell mass in diabetic rats. MP and SP prevented the cognitive dysfunction and glucose dysregulation better than LP. These results suggest that capsaicin might be an effective compound for improving both cognitive dysfuction and hepatic insulin resistance. The results of this study may not be fully applicable to humans since this study was performed in rats infused with β-amyloid into the hippocampus and therefore does not replicate the etiology of human Alzheimer’s disease, although it does closely resemble the pathological features of AD in humans.

## Abbreviations

OGTT: Oral glucose tolerance test
TRPV1: Transient receptor potential vanilloid 1
AD-CON: SUPPLEMENTATION of 1% dextrin supplemented
AD-LP: Supplementation of 1% mild pungency red pepper extract
AD-MP: Supplementation of moderate pungency red pepper extract
AD-SP: Supplementation of sever pungency red pepper
mTOR: Mammalian target of rapamycin
IRS: Insulin receptor substrate
IGF-1: Insulin growth factor-1
Px: Pancreatectomy
HOMA-IR: Homeostasis model assessment for insulin resistance index
PKB or Akt: Protein kinase B
CREB: Camp responding element binding protein
GSK: Glycogen synthase kinase
